# Analgesia in Dentistry*EDITORIAL NOTE.—We are reprinting in the following pages three papers as published in the September *Dental Review*, which were presented to the Chicago Dental Society as a symposium on the subject of Dental Analgesia, by Dr. Wm. H. DeFord, of Des Moines, Iowa, and Drs. W. T. Reeves and E. W. Elliot of Chicago. We have not heretofore seen this subject so comprehensibly and scientifically treated. We commend them to our readers.

**Published:** 1913-09-15

**Authors:** William Harper De Ford

**Affiliations:** Des Moines, Iowa


### ANALGESIA IN DENTISTRY.*

BY WILLIAM HARPER DE FORD, D.D.S., M.D., DES MOINES, IOWA.

Analgesia is a condition of insensibility to pain without the loss of consciousness. The sensation of touch may persist without the sensation of pain Recently in our clinic a

*EDITORIAL NOTE.—We are reprinting in the following pages three papers as published in the September *Dental Review*, which were presented to the Chicago Dental Society as a symposium on the subject of Dental Analgesia, by Dr. Wm. H. DeFord, of Des Moines, Iowa, and Drs. W. T. Reeves and E. W. Elliot of Chicago. We have not heretofore seen this subject so comprehensibly and scientifically treated. We commend them to our readers.

student was operating for a patient with a tooth so sensitive that he simply refused to have the operation completed. Face bathed in perspiration, tears streaming from his eyes, I administered five or six inhalations of somnoform. The student resumed the preparation of the cavity. Patient said: "Wait a moment, I want to tell you something. I can feel the pain just as before, but I have no tendency or desire to dodge or pull away from the instrument." Of course he felt no pain or he would have pulled away just the same as before inhaling the analgesic. Another patient expressed himself in this manner, "I feel the pain just as I did before inhaling that stuff, but I don't care a d—n." I have just said that the sensation of touch may persist without the sensation of pain. These men could feel the pressure of the bur, could hear the sound incident to the cutting, but there was no pain.

One in a state of analgesia hears what is said, knows what is being done, makes reply to questions. Analgesia is the first degree or stage of anesthesia, and differs visibly, mostly in that, in surgical anesthesia the patient is in a condition of profound unconsciousness, while in analgesia the patient knows what is being done, answers questions, follows directions, such as "Open your mouth a little wider," "Turn your head towards me," and makes oral response to such questions as "Do you mind what I am doing," etc.

In *Oral Hygiene*, for January, 1913, I defined a term which I designated Surgical Analgesia.

"Surgical Analgesia implies a state or condition of the patient in which, without complete loss of consciousness, certain surgical procedures may be accomplished without inducing pain; or the pain incident to the operation as ordinarily performed is held in abeyance to such an extent as to elicit no objection on the part of the patient."

This condition which I denominate surgical analgesia varies in different individuals; with some it is present at the very beginning of the analgesic stage, after two or three inhalations; with others it is absent until we approach or reach the beginning of the light anesthesia stage. In the stage even of light anesthesia we have complete loss of consciousness. If the patient is carried this far, we go beyond the analgesia stage and the results are not as satisfactory as when operating a little sooner. It is very desirable that these operations be performed in the stage of analgesia rather than that of light anesthesia. In the stage of light anesthesia respiration is considerably deeper and quicker than normal, and the heart's action excited, hence patients are more apt to become excited, and having passed into unconsciousness, cannot assist the operator to the extent that a patient can who responds to such demands as "Turn your head towards me," "Elevate your chin," etc. Either operate in the stage of surgical analgesia or push on to the stage of surgical anesthesia.

The analgesic stage is ushered in by a sense of warmth or mild stimulation, because of the fact that there is a slight rise of blood pressure and slightly increased respiration, conditions conducive to safety. More patients quickly recognize a feeling of numbness in the lower extremities extending to the hands, cheeks, lips, gums, and upon snapping the teeth together, a sense of deadness, and they frequently volunteer the expression, "I know you can now operate with out my feeling pain, and even extract teeth." In nearly all cases patients will indicate the stage at which they believe painless operating can be done, and after once having experienced this sensation will, at future sittings accurately estimate the moment at which the operation can begin. Very seldom are they mistaken as to when it is safe to begin operating, and the fact that they feel that painless cavity preparation can be made at that particular time, exerts a helpful psychic influence, very much to be desired by the operator. I sometimes think the patient, feeling himself passing away, not wishing to lose consciousness, takes a chance of being in the proper condition, and surprisingly finds that it is so.

This sense of stimulation is to most patients very agreeable. A month ago I happened to step into the office of the dean of one of our Southern dental colleges. He introduced me to a patient to whom had been administered somnoform for analgesia induction, the operation in question being that of the removal of serumal deposits in a case of advanced pyorrhea. I inquired of this patient as to an expression on her part of the difference in feeling at first without the aid of an anesthetic and then under analgesia, and she replied, "I could have but two teeth operated at a sitting without the anesthetic, and it was extremely painful, but in the analgesic state six teeth, all upper molars, were operated at a sitting without a particle of pain and the sensation experienced was positively pleasant."

There is this about analgesia operations. A patient once submitting to this method will never again allow you to operate for him in painful conditions without it.

We aim to have each of our students do a certain number of operations under analgesia to familiarize themselves with the technic, and these patients returning, finding that they must submit to other work without the aid of the analgesia have refused to have their work completed, saying they would go to a private office where this method could be used.

The new beginner need not become discouraged if he is not entirely successful at first, bearing in mind that even if he does not succeed in inducing an ideal analgesia, a partial failure is better than no application at all.

All general anesthetic agents have their analgesic stage. Analgesia can be induced by inhalation of nitrous oxid and oxygen, nitrous oxid and air, somnoform, ether, chloroform, ethyl chlorid, ethyl bromid, and other agents not so generally known, but for dental purposes I believe that nitrous oxid and somnoform are to be preferred above all other general anesthetics for analgesia induction.

Safety is the first requisite in selecting an analgesic agent, and statistics show that in the matter of safety nitrous oxid and somnoform are far less dangerous than other general anesthetics. The death rate for these anesthetics is only one in several hundred thousand administrations. With such a record for nitrous oxid and somnoform for cases of surgical anesthesia, how much safer these agents must be when used only in the analgesic stage. And bear in mind please, that these figures include nitrous oxid and somnoform administrations from the very beginning when the appliances were most crude and unscientific, and those using them usually had no previous knowledge of anesthetics, relying for the most part upon the printed instructions that accompanied the appliance.

Other things being equal, the anesthetic agent pleasant to inhale, most free from disagreeable effects during and after administration is the one to be preferred. With nitrous oxid and somnoform, by means of modern appliances these gases can be administered so gently as to be almost undetected. Should these however, prove objectionable to the fastidious, or the elite, or the patient imagine them so, a few drops of essence of orange will completely disguise the aroma of these agents, and even the disagreeable, penetrating, nauseating ether odor can be completely covered by a little essence of orange.

Easy and rapid production of effect, when this can be done safely and easy and rapid recovery are greatly to be desired. With nitrous oxid and somnoform, deep anesthesia can be induced in from thirty to forty-five seconds, and usually in a minute or two after returning to consciousness all effects of the anesthetic are gone.

Simplicity of administration is a most important feature. Nitrous oxid is a good anesthetic, and it will accomplish all that is claimed for it and failure results from its use simply because men have not the patience, skill and perseverance to stay by it till they learn how. Analgesia cannot be induced by pure nitrous oxid for surgical purposes without the admixture of air or oxygen, and when we add oxygen the process becomes even more complicated. We now have to deal with two gasses instead of one, and the proportions must be preserved and maintained, and the pressure behind them must be constant, but it is time well spent to learn this accomplishment. Somnoform is the easiest of all anesthetics to administer. Men who make most dismal nitrous oxid failures, experience little or no difficulty in inducing somnoform anesthesia or analgesia. Somnoform appliances are very much less complicated than nitrous oxid appliances, much easier to manipulate, weigh less than a pound, no cylinders, pressure gauges, or warming devices are necessary, and the somnoform comes in convenient glass capsules, and can be sent by means of parcel post. Somnoform anesthesia and analgesia can be induced without the admixture of oxygen; air dilution is all that is necessary, and cases are on record of sixty-minute somnoform analgesia with one-eighth somnoform and seven-eighths air, the patient at no time losing consciousness, no distressing or alarming symptoms and entire absence of pain.

Although I induce analgesia just as patients happen to come and have witnessed nausea only a few times with nitrous oxid and not at all with somnoform, in private practice. I believe it best to make appointments two or three hours after the patient has eaten a meal, or have them abstain from food. In the case of women the corset and all bands should be loosened, the neck muscles should be free from pressure with all patients, and the bladder should be emptied before taking the chair.

It is prohibitive to consider analgesic technic in a twenty-minute paper, and I will not attempt it.

We operate on vital tissue, "perform laparotomies on the teeth," so to speak, the patient suffers, cringes, agonizes, almost to the point of collapse and the anesthetic usually employed is that of witty speech or an amusing story.

If pain meant no more than the hurt at the time, and we could properly cut down to healthy dentin, and could obtain correct cavity preparation, and could make the patient submit to all necessary cutting no matter how severe, it might be excusable to continue present methods, but pain intense in its character and even when slight if long continued is detrimental to our well being, shatters the nerves, induces neurasthenia, breaks down the will power and enervates our physical being. "Exhaustion of the vaso-motor centers rather than structural lesions is what produces shock, and I want to emphasize the fact that it is a dangerous procedure to submit even the physically strong, more so the frail, to intense pain beyond certain limits."[3] It seems almost beyond comprehension, when the means is at hand to to prevent pain, and the public are clamoring for it, demanding it and will pay any price to obtain it, we go on in the same old way obtaining inferior results, knowing at the time that the operation falls short of what it should be, and will fail in a few months because we cannot remove the carious tissue thoroughly or cannot prepare a cavity sufficiently well for the retention of the filling or inlay, and fail utterly in shaping a tooth for a crown because it becomes too sensitive and are defeated in removing calcarious deposits, yet 95 per cent. of dental surgeons continue this line of practice.

I do not believe there is a man within the hearing of my voice, except those that operate under analgesia, that does not every day fall short of doing what he knows would be better for his patients. We adjust the rubber dam, map out in our minds the cavity preparation we believe best for a particular operation, break down enamel walls with chisels, remove soft decay with excavators, resort to the bur for the removal of less carious dentin and proper shaping of the tooth for retention of filling or inlay, but the tooth becomes extremely sensitive, we cut in a less sensitive area, finally it is alike sensitive in all directions, yet some carious dentin remains and the cavity is not the shape that will retain the filling or inlay. Patient is becoming more nervous all the time, the amount of pain well borne in the earlier part of the operation is almost unbearable now. Contortions of the face and restlessness, pulling away from the bur, together with verbal protests such as, "I am doing my best, Doctor,

---

3. Lectures on General Anesthetics in Dentistry, Second Edition, page 22.

but I just cannot stand this any longer." We begin to say to ourselves, now if I can get enough retention to retain the filling, I will let that discoloration go, it will probably make no difference. In this way we compromise cavity preparation, and we fail to remove all carious dentin. Carious dentin bears the same relation to dentistry as carcinoma does to surgery, and the dental surgeon is as culpable who overlooks this condition as the general surgeon who does not do a radical operation in cancerous conditions. We can no more do our work as thoroughly as it should be done, than the general surgeon can do his work properly without employing every means available.

This movement is spreading rapidly. Prominent dental surgeons all over the country are becoming interested in analgesia. As a dental educator there is no more forceful or influential man in the dental profession than Dr. H. E. Friesell dean of the University of Pittsburg, College of Dentistry. This past year he was president of the Institute of Dental Pedagogics. In a letter received from him just recently he says, "When my address as presented to the Institute of Dental Pedagogics comes out you will see that I have laid stress therein upon the fact that dental schools must devise and teach safe and certain methods of anesthesia and analgesia in order to eliminate pain absolutely from dental operations. This is exactly what I feel in the matter and no one can do a more important service to humanity and dentists than to bring about this condition. I do not mean to teach a few students who are especially interested to accomplish this, but to teach every student, so that within a few years any pain whatever in connection with a dental operation will be little short of mal-practice. This is one of the problems dentists must solve." I have never dared go so far; I have never written or spoken as plainly as Dr. Friesell, but he has expressed exactly what I have believed and hoped for these many years.

